# A comparison of diagnostic algorithms and clinical parameters to diagnose ventilator-associated pneumonia: a prospective observational study

**DOI:** 10.1186/s12890-021-01527-1

**Published:** 2021-05-13

**Authors:** Farshid Rahimibashar, Andrew C. Miller, Mojtaba H. Yaghoobi, Amir Vahedian-Azimi

**Affiliations:** 1 Anesthesia and Critical Care Department, Hamadan University of Medical Sciences, Hamadan, Iran; 2 Department of Emergency Medicine, Nazareth Hospital, Philadelphia, PA USA; 3 Department of Infectious and Tropical Diseases, Alborz University of Medical Sciences, Alborz, Iran; 4 Trauma Research Center, Nursing Faculty, Baqiyatallah University of Medical Sciences, Tehran, Iran

**Keywords:** Ventilator-associated pneumonia, Cross infection, Artificial respiration, Critical care

## Abstract

**Background:**

Suspicion and clinical criteria continue to serve as the foundation for ventilator-associated pneumonia (VAP) diagnosis, however the criteria used to diagnose VAP vary widely. Data from head-to-head comparisons of clinical diagnostic algorithms is lacking, thus a prospective observational study was performed to determine the performance characteristics of the Johanson criteria, Clinical Pulmonary Infection Score (CPIS), and Centers for Disease Control and Prevention’s National Healthcare Safety Network (CDC/NHSN) criteria as compared to Hospital in Europe Link for Infection Control through Surveillance (HELICS) reference standard.

**Methods:**

A prospective observational cohort study was performed in three mixed medical-surgical ICUs from one academic medical center from 1 October 2016 to 30 April 2018. VAP diagnostic criteria were applied to each patient including CDC/NHSN, CPIS, HELICS and Johanson criteria. Tracheal aspirate cultures (TAC) and serum procalcitonin values were obtained for each patient.

**Results:**

Eighty-five patients were enrolled (VAP 45, controls 40). Using HELICS as the reference standard, the sensitivity and specificity for each of the assessed diagnostic algorithms were: CDC/NHSN (Sensitivity 54.2%; Specificity 100%), CPIS (Sensitivity 68.75%; Specificity 95.23%), Johanson (Sensitivity 67.69%; Specificity 95%). The positive TAC rate was 81.2%. The sensitivity for positive TAC *with* the serum procalcitonin level > 0.5 ng/ml was 51.8%.

**Conclusion:**

VAP remains a considerable source of morbidity and mortality in modern intensive care units. The optimal diagnostic method remains unclear. Using HELICS criteria as the reference standard, CPIS had the greatest comparative diagnostic accuracy, whereas the sensitivity of the CDC/NHSN was only marginally better than a positive TAC plus serum procalcitonin > 0.5 ng/ml. Algorithm accuracy was improved by adding serum procalcitonin > 0.5 ng/ml, but not positive quantitative TAC.

*Trial Registration*: Not indicated for this study type.

## Background

The incidence of nosocomial infections (NI) amongst intensive care unit (ICU) patients is 2–5 times that of general admissions [1]. Amongst the most prevalent and threatening ICU NIs is ventilator-associated pneumonia (VAP), which may develop in patients receiving invasive mechanically ventilated (MV) for ≥ 48 h [2–6]. VAP has a cumulative incidence of 10–45%, and an attributable risk of 5–27% [7–12]. VAP-associated comorbidities include prolonged duration of MV, delayed MV weaning, increased antibiotic consumption, prolonged ICU and hospital length-of-stay (LOS), increased treatment-related expenditures, and increased crude and attributed mortality with recent studies reappraising the impact of VAP on mortality to be 10% [2–6, 13–17]. Accordingly, VAP prevention has emerged as a high priority. As such, one component of the *Institute for Healthcare Improvement’s* recommended ventilator bundle is the accurate diagnosis and determination of VAP incidence [18–20]. However, the optimal VAP diagnostic strategy remains contentious. Research in this field is limited by the lack of a consensus ‘gold standard’ definition against which to test the diagnostic accuracy of new diagnostic algorithms or methods of detection. VAP diagnosis remains challenging as clinical signs and symptoms may be non-specific, with clinical diagnosis being overly sensitive (leading to increased antibiotic use), and histopathology (ante- or post-mortem within 96 h of death) being limited in availability, consistency, standardization and reliability [21–23]. Moreover, quantitative respiratory cultures have been found to correlate poorly with histopathology [22, 24].

As none of the available diagnostic tests, performed alone, can provide an accurate diagnosis of VAP, a diagnostic strategy incorporating several criteria has been viewed by many to be a good compromise. To this end, great effort has been expended to generate standardized diagnostic algorithms that incorporate clinical, radiographic and microbiological data. Some examples (Table 1) include: Centers for Disease Control and Prevention’s National Healthcare Safety Network (CDC/NHSN) [25], Clinical Pulmonary Infection Score (CPIS) [26], Hospital in Europe Link for Infection Control through Surveillance (HELICS) [27], Johanson criteria [28], and others [29, 30]. As compared to immediate post-mortem lung biopsies, clinical criteria have reasonable diagnostic performance but may be highly impacted by the diagnostic thresholds used, and the lack of a uniform reference diagnostic standard has contributed to variable diagnostic performance (Table 2) and made inter-study comparisons difficult [31]. A highly performing VAP diagnostic method is greatly needed, but international guidelines disagree on the use of clinical algorithms for risk stratification to determine treatment [32, 33]. Data comparing algorithm performance head-to-head is lacking, and as most such data stems from high-income countries. Great need exists for head-to-head comparisons, as well as data from low-to-middle income countries to supplement the international data pool. To this end, a prospective non-randomized study was conducted to determine if in patients with VAP, does application of the CDC/NHSN, CPIS, or Johanson criteria provide the greatest diagnostic performance characteristics as compared to HELICS as the reference standard.

**Table 1:** Ventilator-associated pneumonia diagnostic algorithms utilized in this study

Published Criteria (citation)	Systemic Criteria	Chest Criteria	Chest Radiography Criteria	Microbiologic Criteria
CDC/NHSN (25)	- Inflammatory response Temperature > 38 °C WBC > 12,000/mm^3^ or < 4,000/mm^3^ - OR new antimicrobial agent is started for ≥ 4 days → Infection-related ventilator-associated complication	After a period of stability or improvement on the ventilator (≥ 2 calendar days of stable or ↓ F_i_O_2_ or PEEP): - Minimum daily F_i_O_2_ ↑ ≥ 0.20 lasting 2 days - Or minimum daily PEEP values ↑ ≥ 3 cm H_2_O lasting 2 days → Ventilator-associated condition	–	Microbiologic quantitative ( +), OR histologic ( +), OR ( +) for legionella, influenza, RSV, adenovirus, or parainfluenza virus AND Gram-stain evidence ≥ 25 neutrophils/lpf and ≤ 10 epithelial cells/lpf → Probable VAP
CPIS^a^ (26)	Fever: - 38.5–38.9 (1 point) - ≥ 39 or < 36.5 (2 points) WBC: - < 4,000/mm^3^ or > 11,000/mm^3^ (2 points)	- Secretions but not purulent (1 point) - Purulent secretions (2 points) - P_a_O_2_/ F_i_O_2_ < 240 without ARDS (2 points)	Diffuse infiltrate (1 point) Localized infiltrate (2 points) Progressive infiltrate (without cardiac disease or ARDS) (+ 2)	Positive (1 point)
HELICS^b^ (27)	At least 1 criterion: - Temperature > 38 °C (with no other cause) - WBC > 12,000/mm^3^ or < 4,000/mm^3^ - If age > 70 years: AMS without other cause	At least 1 of following criteria (2 clinical pneumonia only = PN4 and PN5): New onset purulent sputum or change in sputum character (color, odor, quantity, consistency) Cough or dyspnea or tachypnea Suggestive auscultation (rales or bronchial breath sounds, rhonchi, wheezing) Worsening gas exchange (O_2_ desaturation, increasing F_i_O_2_ requirements or ventilation demands)	Image suggestive of pneumonia. (≥ 2 serial chest X-rays or CT scans with suggestive imaging for patients with underlying cardiac or pulmonary disease)	PN1 – ( +) quantitative Cx from minimally contaminated LRT specimen ^c^ PN2 – ( +) quantitative Cx from possibly contaminated LRT specimen ^d^ PN3 – Alternative methods: ^e^ ( +) blood or pleural Cx, pleural or pulmonary abscess, histology, or pathogen antigen or antibody testing PN4 – ( +) sputum Cx or non-quantitative LRT specimen Cx PN5 – No positive results
Johanson (28)	Temperature > 38.5 °C WBC > 12,000/mm^3^	Purulent secretions	New or progressive consolidation	–

AMS: altered mental status; ARDS: acute respiratory distress syndrome; CDC/NHSN: centers for disease control and prevention national health safety network; CPIS: clinical pulmonary infection score; Cx: culture; F_i_O_2_: fraction of inspired oxygen; HELICS: hospital in Europe link for infection control through surveilance; LRT: lower respiratory tract; P_a_O_2_: partial pressure of oxygen in arterial blood; RSV: respiratory syncytial virus; VAP: ventilator associated pneumonia; WBC: white blood cell

^a^Score > 6 is suggestive of VAP

^b^VAP diagnosis if criteria met and invasive respiratory device (even intermittently) in the 48 h preceding the onset of infection

^c^Either: (1) Broncho-alveolar lavage (BAL) with a threshold of ≥ 104 cfu/mL or ≥ 5% of BAL obtained cells contains intracellular bacteria on direct microscopic exam; (2) Protected brush (PB Wimberley) with a threshold of ≥ 103 cfu/mL; (3) Distal protected aspirate (DPA) with a threshold of ≥ 103 cfu/mL

^d^Quantitative culture of LRT specimen (e.g., endotracheal aspirate) with a threshold of 106 cfu/mL

^e^Either: (1) positive blood culture not related to another source of infection; (2) positive growth in culture of pleural fluid; (3) pleural or pulmonary exam shows evidence of pneumonia; (4) positive exams for pneumonia with virus or particular germs (Legionella, Aspergillus, mycobacteria, Mycoplasma, Pneumocystis carinii). The latter may include: (A) positive detection of viral antigen or antibody from respiratory secretions (e.g., EIA, FAMA, shell vial assay, PCR); (B) positive direct exam or positive culture from bronchial secretions or tissue; (C) seroconversion (ex: influenza viruses, Legionella, Chlamydia); or (D) detection of antigens in urine (Legionella)

**Table 2:** Performance characteristics of ventilator-associated pneumonia diagnostic algorithms

Criteria Studied	Year, (citation)	Population	Comparator	Sample Size	Sensitivity	Specificity	PPV	NPV	( +) LR	(-) LR	ROC AUC	Kappa (ĸ) index, agreement level ^a^
CDC/NHSN	2015, (60)	Mixed^b^	CPIS	38	0.37	1.0	1.0	0.84				ĸ = 0.47, moderate
CPIS	1999, (43)	Mixed^b^	Pathology	23	0.77	0.42						
CPIS^c^	2004, (66)	Mixed^b^	Quantitative Cultures	69	0.41	0.77	0.8	0.36			0.64	
CPIS	2004, (67)	Mixed^b^	Quantitative Cultures	88	0.89	0.47	0.57	0.84				ĸ = 0.33, fair
CPIS	2007, (68)	Burn	Quantitative Cultures	28	0.30	0.80	0.70	0.50				
CPIS	2010, (40)	Mixed^b^	Pathology	142	0.46	0.60			1.13	0.96		
CPIS	2015, (69)	Surgical (mixed)	Quantitative Cultures	497	0.633	0.644	0.61	0.674			0.60	
CPIS	2018, (70)	Surgical (acute care)	Quantitative Cultures	198	0.611	0.781	0.64	0.759				
HELICS	2013, (36)	Mixed^b^	Not clearly specified	57^d^	0.86	0.99	0.77	0.995				ĸ = 0.80, substantial
Johansen^e^	1999, (43)	Mixed^b^	Pathology	23	0.69	0.75						
Johansen	2018, (70)	Surgical (acute care)	Quantitative Cultures	198	0.828	0.59	0.564	0.843				
NTDB/NTR	2015, (71)	Trauma	CDC/NHSN	279	0.864	0.578	0.74	0.74				ĸ = 0.47, moderate

CDC/NHSN: centers for disease control and prevention national health safety network; ICU: intensive care unit; MV: mechanical ventilation; NR: not reported; NTDB/NTR: national trauma data bank / national trauma registry; NPV: negative predictive value; PPV: positive predictive value; ROC AUC: receiver operating curve area under curve; ( +) LR: positive likelihood ratio; (-) LR: negative likelihood ratio

^a^Agreement based on score: ≤ 0 (no agreement); 0.01–0.20 (slight); 0.21–0.40 (fair); 0.41– 0.60 (moderate); 0.61–0.80 (substantial); and 0.81–1.00 (almost perfect agreement)

^b^A mixed population containing both medical and surgical patients. Studies that did not specify ICU type were by default classified as mixed

^c^For CPIS threshold of > 7, rather than current standard of > 6. The AUC using threshold CPIS > 6 was 0.54, other values not reported

^d^Data from sub-population of a larger study assessing various types of ICU-acquired infections

^e^The presence of all three criteria increased the specificity to 92% at the cost of a high beta error (sensitivity 23%)

## Methods

A prospective observational cohort study was performed in three mixed medical-surgical ICUs from one academic medical center from 1 October 2016 to 30 April 2018. The study was approved by the Investigational Review Board at Hamadan University of Medical Sciences, Hamadan, Iran (IR.UMSAHA.REC.1395.23). All study parts were reviewed according to the *Strengthening the Reporting of Observational Studies in Epidemiology ‘STROBE’* guideline [34]. Written consent was required and covered both study participation and publication of de-identified aggregate findings. Surrogate consent from the patient’s legal guardian or designated health proxy was permitted in cases where the subject lacked decision-making capacity. All patients that survived and regained their faculties were informed of the project. All data generated or analyzed during this study are included in this article. De-identified individual subject data may be available from the corresponding author on reasonable request.

**Fig. 1:**
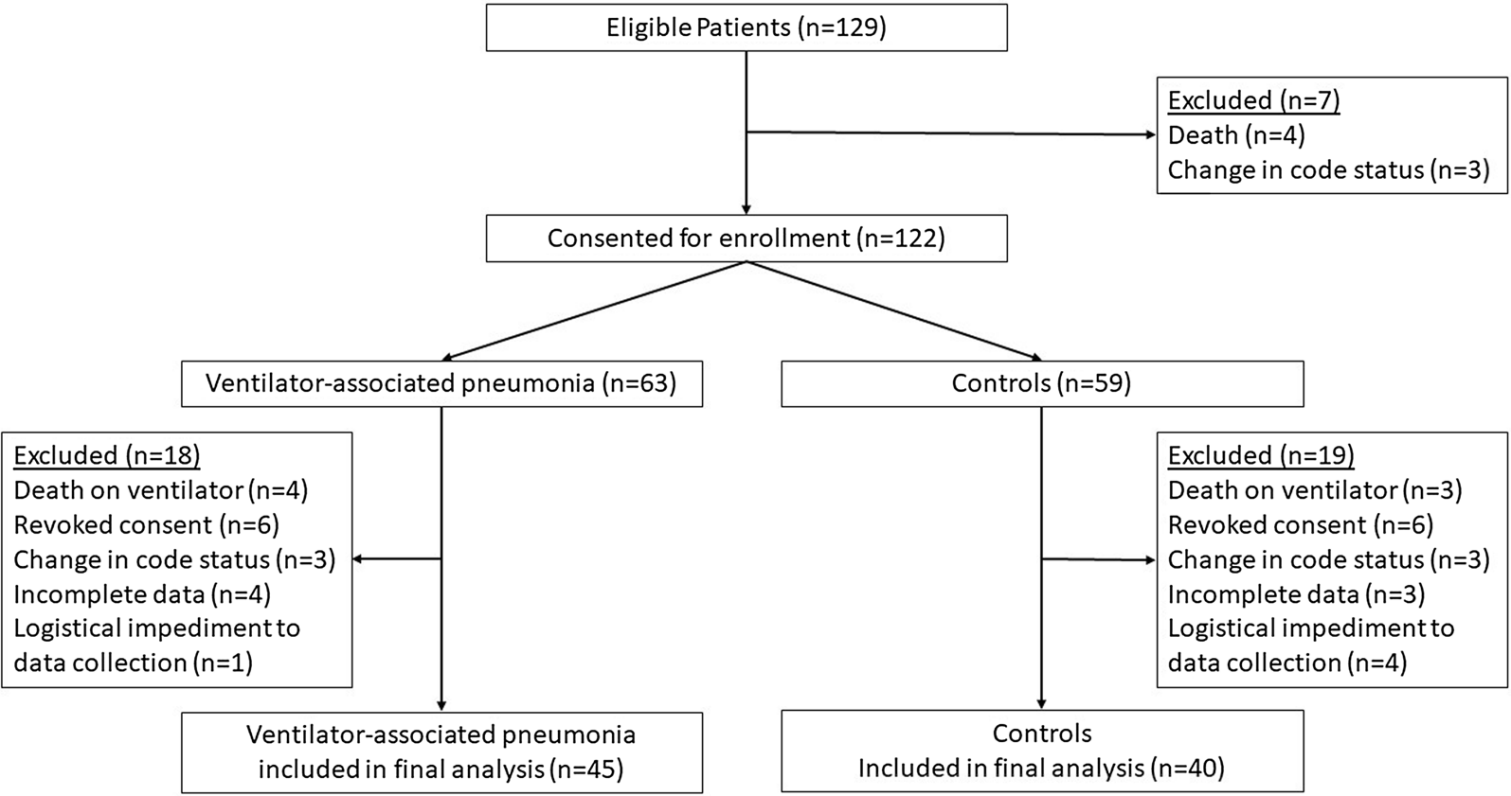
Patient flow diagram

Patients were eligible for study participation if: (1) age ≥ 18 years, (2) admitted to the ICU > 48 h, (3) receiving invasive MV > 48 h (any mode except high frequency percussive ventilation or high frequency oscillatory ventilation), (4) full-code status, and (5) informed consent obtained from the patient, legal guardian or healthcare surrogate upon ICU admission (prior to intubation). Patients with any limitation of code status including (but not limited to) *No Code, Do Not Resuscitate,* or *Do Not Intubate* were excluded (Fig. 1). Patients with known pregnancy were excluded.

Patient selection was performed by an enrollment team of two physicians (1 critical care, 1 infectious disease) not directly involved in the study. All consecutive patients identified at the participating ICUs with VAP according to the HELICS criteria were eligible. Each case patient was matched by the enrollment team, which was blinded to the outcome, with another ICU patient that did not have VAP. Matching was based on: (1) admission indication; (2) ICU LOS ≥ 48 h; (3) receiving invasive MV > 48 h (any mode except high frequency percussive ventilation or high frequency oscillatory ventilation as these preclude proper calculation of the CBC/NHSN criteria); (4) severity of illness at ICU admission as quantified by the Acute Physiology and Chronic Health Evaluation (APACHE) II score > 15, (5) full code status, and (6) age ≥ 18 years.

VAP diagnosis was made independently by the treating clinical team. Diagnostic criteria were according to HELICS criteria [27] in accordance with the institutional standard and other published studies [2, 35–38] as it is the definition currently used in much of Europe, Australia, and the near- and middle east (including Iran). Chest radiograph interpretation was undertaken “off-line” by a team of 3 physicians (1 radiology, 1 critical care, 1 pulmonology) who were independent of the treating team. Kendal agreement coefficient between the clinicians in chest radiograph interpretation was 0.99. Procalcitonin was measured at the time of initial VAP suspicion. A single value was used, and thresholds were in accordance with prior published studies [39].

### Specimen collection and processing

Protected tracheal aspirate (TA) samples were obtained through a sterile 12 French catheter (SUPA Medical Devices, Tehran, Iran). This catheter is placed in the trachea by advancing through the endotracheal tube until resistance was encountered (level of the carina) and retracted approximately 2 cm. To obtain TA samples, 5–10 mL of sterile saline was instilled followed by aspiration into a sterile syringe. This generally yielded an aspirate of 2-3 cc. The samples were then transferred to the microbiology laboratory for processing and examination within 30 min. The materials were evaluated by gram-stain and quantitative cultures. Light microscopy was utilized to assess gram stains for bacteria and white blood cells. The samples were vortexed for one minute at 3,000 rpm, diluted with saline to 1:10 ratio, and 0.01 cc inoculated onto blood agar, chocolate agar, and MacConkey agar plates. Cultures were incubated at 35 ± 1ºC for 24, followed by quantitative bacterial evaluation. The cut-off values for bacterial colony counts were taken as ≥ 105 colony forming units (CFU)/cc. When more than one bacteria type was identified, a separate colony count was performed for each. Microbial identification and antimicrobial susceptibility testing were performed using the automated Vitek® 2 Advanced Expert System (bioMérieux, Marcy-l'Étoile, France).

The criteria for sample rejection were: (1) improperly labeled specimens, (2) specimens with transport times exceeding study standards, (3) clotted specimens, (4) specimens not submitted in an appropriate transport container, (5) insufficient volume, or (6) external contamination. If an unacceptable specimen was received, the treatment team was notified, and another specimen was requested.

### Data collection

Screening, data collection and reporting was undertaken by a trained, dedicated full-time nurse. The data collection tool was a two-part checklist including demographic variables, clinical and microbiological variables. The tool was developed during two 90-min meetings by a consensus multidisciplinary panel consisting of 17 physicians representing critical care (n = 5), anesthesia (n = 3), pulmonology (n = 5), internal medicine (n = 3), and forensic medicine (n = 1), and 10 critical care nurses. The Quantitative face validity was determined using Impact Score (2.5–4.5), and quantitative content validity was determined via 27 panelists. The measured content validity ratio and content validity index were 0.51 and 0.89 respectively. The internal validity of the questionnaire was determined by Cronbach's alpha coefficient to be 0.91.

### Statistics

Statistical analyses were performed using IBM® SPSS version 22.0 (IBM Corp, Armonk, USA). Data were summarized using mean ± standard deviation (SD) for quantitative variables and frequency (%) for qualitative variables. Study size was determined by *a prior* sample size calculation. Considering a VAP prevalence of 0.5, 95% confidence interval level, 80% power, and absolute error 10%, the necessary sample size was calculated to be 85 patients.

Normally distributed variables were compared using the Student’s t-test. Categorical variables were compared using Chi-square (χ^2^) test or Fisher's exact test when appropriate. Trend of change in distribution of relative frequencies between ordinal data were compared using χ^2^ test for trend. The Youden index (or Youden’s J Statistic) was calculated as: *J* = *sensitivity* + *specificity – *1.

## Results

One-hundred twenty-nine patients were screened, and 85 were included in the final analysis (Fig. 1). The mean age was 46.94 ± 18.90 years with a male predominance (72.9%). Measures of illness severity and hospital course metrics are listed in Table 3. Positive tracheal culture was seen in 81.2% with cultures yielding Acinetobacter (37.6%), Staphylococcus aureus (22.4%), Escherichia coli (14.1%), Pseudomonas (10.6%), Klebsiella (10.6%), and Proteus (3.5%). Multiple drug resistant (MDR) organisms were identified in 36.5% of isolates. The sensitivity and specificity of the tested algorithms are presented in Table 4. Of note, the sensitivity for positive TAC *with* the serum procalcitonin level > 0.5 ng/ml was 51.8%, lower than each of the algorithms assessed. The highest Youden index, a measure of diagnostic accuracy, was seen with CPIS (Table 4).

**Table 3:** Patient demographic and clinical information

Variable	All	VAP n = 45	No VAP n = 40	p-value
Age, years, mean (SD)	46.9 (18.9)	44.2 (20.7)	49.9 (16.4)	0.159^a^
Male, N (%)	62 (72.9)	33 (73.3)	29 (72.5)	0.931^c^
*Admission indication, N (%)*				0.652^c^
Trauma	54 (63.5)	30 (66.7)	24 (60)	
Post-operative	31 (36.5)	15 (33.3)	16 (40)	
*Comorbidities, yes, N (%)*				0.932^b^
ARDS	7 (8.2)	3 (6.7)	4 (10)	
Cancer	13 (15.3)	6 (13.3)	7 (17.5)	
COPD	7 (8.2)	4 (8.9)	3 (7.5)	
CHF	24 (28.2)	13 (28.9)	11 (27.5)	
ESRD	14 (16.5)	9 (20)	5 (12.5)	
Multiple trauma	20 (23.5)	10 (22.2)	10 (25)	
Positive tracheal culture, N (%)	69 (81.2)	40 (88.9)	29 (72.5)	0.093^c^
MDR organism, yes, N (%)	31 (36.5)	17 (37.8)	14 (35)	0.825^c^
Procalcitonin, ng/mL, mean (SD)	4.03 (4.68)	3.53 (3.6)	4.6 (5.6)	0.308^a^
APACHE II, mean (SD)	18.1 (2.84)	17.9 (3.43)	18.4 (1.98)	0.399^a^
Duration of intubation, hours, mean (SD)	177.1 (39.61)	176.02 (38.7)	178.32 (41.09)	0.791^a^
Reintubation, N (%)	32 (37.6)	14 (31.1)	18 (45)	0.262^c^
MV duration prior to VAP, hours, median (IQR)	72 (54–87.5)	72 (52–87.5)	72 (64.5–88.5)	0.639^a^
ICU duration prior to developing VAP, days, median (IQR)	7 (6–8)	7 (6–8.5)	7 (6–8)	0.118^a^
*VAP timing, mean (SD)*				
Early (< 5 days)	–	15 (33.3)	–	–
Late (≥ 5 days)		30 (66.7)		
*Length-of-stay, days, mean (SD)*				
ICU LOS	9.8 (3.0)	13.13 (3.27)	12.72 (2.75)	0.538^a^
Non-ICU LOS	15.4 (3.1)	12.67 (3.34)	11.96 (2.99)	0.320^a^
*Mortality, N (%)*				
ICU	17 (20)	8 (17.8)	9 (22.5)	0.787^c^
Hospital	22 (25.9)	12 (26.7)	10 (25)	0.861^c^

VAP: ventilator-associated pneumonia; IQR: interquartile range; MDR: multiple drug resistant; APACHE: Acute Physiology and Chronic Health Evaluation; ICU: intensive care unit; VAP: ventilator-associated pneumonia; LOS: length-of-stay

^a^Independent sample t-test

^b^Fisher exact test

^c^Chi-square

**Table 4:** Sensitivity, specificity, and Youden index for assessed methods of ventilator-associated pneumonia diagnosis compared to the HELICS criteria as the reference standard

Criteria	Ventilator-Associate Pneumonia			% Sensitivity	% Specificity	Youden index ^a^
	Positive	Negative	Total			
*CDC/NHSN*						
Positive	45	38	83	54.22	100	0.542
Negative	0	2	2			
Total	45	40	85			
*CPIS*						
Positive	44	20	64	68.75	95.23	0.640
Negative	1	20	21			
Total	45	40	85			
*Johanson*						
Positive	44	21	65	67.69	95	0.627
Negative	1	19	20			
Total	45	40	85			

CDC/NHSN = centers for disease control and prevention national health safety network; CPIS = Clinical Pulmonary Infection Score, HELICS = Hospital in Europe Link for Infection Control through Surveillance

^a^A measure of the maximum diagnostic accuracy, where 1 signifies a perfect test and 0 signifies no diagnostic value

The Kappa agreement coefficient results between each diagnostic algorithm and either serum procalcitonin level or positive TAC is highlighted in Table 5. The greatest correlation between positive VAP assessment and serum procalcitonin levels > 0.5 ng/ml was observed with the Johanson method and CPIS (both roughly 70%).

**Table 5:** Correlation of serum procalcitonin and tracheal aspirate results with ventilator-associated pneumonia diagnostic algorithms

Criteria	Serum Procalcitonin Level, ng/mL				Kappa (ĸ) index, agreement level ^a^ (p-Value)	Tracheal Culture			Kappa (ĸ) index, agreement level ^a^ (p-Value)
	< 0.25	0.25–0.5	> 0.5	Total		Positive	Negative	Total	
*Johanson, n (%)*									
Positive	10 (15.4)	9 (13.8)	46 (70.8)	65	0.47, moderate (< 0.001)	61 (93.8)	4 (6.2)	65	0.579, moderate (< 0.001)
Negative	18 (90)	0	2 (10)	20		8 (40)	12 (60)	20	
Total	28 (32.9)	9 (10.6)	48 (56.5)	85 (100)		69 (81.2)	16 (18.8)	85 (100)	
*CDC/NHSN, n (%)*									
Positive	26 (31.3)	10 (12.0)	47 (56.6)	83	0.06, slight (0.58)	67 (80.7)	16 (19.3)	83	0.04, slight (0.49)
Negative	2 (100)	0	0	2		2 (100)	0	2	
Total	28 (32.9)	10 (11.8)	47 (55.3)	85 (100)		69 (81.2)	16 (18.8)	85 (100)	
*CPIS, n (%)*									
Positive	11 (17.5)	8 (12.7)	44 (69.8)	63	0.42, moderate (< 0.001)	61 (96.8)	2 (3.2)	63	0.663, substantial (< 0.001)
Negative	17 (77.3)	1 (4.5)	4 (18.2)	22		8 (36.4)	14 (63.6)	22	
Total	28 (32.9)	9 (10.6)	48 (56.5)	85 (100)		69 (81.2)	16 (18.8)	85 (100)	

CDC/NHSN: centers for disease control and prevention national health safety network; CPIS: Clinical Pulmonary Infection Score, HELICS: Hospital in Europe Link for Infection Control through Surveillance

^a^Agreement based on score: ≤ 0 (no agreement); 0.01–0.20 (slight); 0.21–0.40 (fair); 0.41– 0.60 (moderate); 0.61–0.80 (substantial); and 0.81–1.00 (almost perfect agreement)

As stated previously, CPIS correlated most closely with the HELICS standard. However, when comparing the three tested algorithms, CPIS displayed near perfect agreement with the much simpler and historical Johanson criteria, whereas CDC/NHSN showed only slight agreement with either of the other algorithms (Table 6). Moreover, CPIS correlated most closely with traditional clinical markers for pneumonia (Table 7).

**Table 6:** Kappa agreement coefficient among ventilator-associated pneumonia diagnostic methods

Criteria	Kappa (ĸ) index, agreement level ^a^	p-Value
CPIS and Johanson	0.874	< 0.001
CDC/NHSN and Johanson	0.145	< 0.001
CDC/NHSN and CPIS	0.129	0.015

CDC/NHSN: centers for disease control and prevention national health safety network; CPIS: Clinical Pulmonary Infection Score, HELICS: Hospital in Europe Link for Infection Control through Surveillance

^a^Agreement based on score: ≤ 0 (no agreement); 0.01–0.20 (slight); 0.21–0.40 (fair); 0.41– 0.60 (moderate); 0.61–0.80 (substantial); and 0.81–1.00 (almost perfect agreement)

**Table 7:** Correlation of individual variables with ventilator-associated pneumonia diagnostic methods

Parameter	Kappa agreement coefficient		
	CDC/NHSN	CPIS	Johanson
PCT > 0.5 ng/ml	0.061	0.423	0.470
Infiltrate on radiograph	− 0.045	0.874	0.738
Temperature	− 0.044	0.529	0.579
WBC	− 0.044	0.739	0.729
P_a_O_2_	− 0.038	0.094	-0.139
Tracheal culture	0.044	0.663	0.579
Blood culture	− 0.011	0.238	0.165

CDC/NHSN: centers for disease control and prevention national health safety network; CPIS: Clinical Pulmonary Infection Score, HELICS: Hospital in Europe Link for Infection Control through Surveillance, PCT: Serum procalcitonin; WBC: White blood cell; P_a_O_2_: Partial pressure of O_2_ in arterial blood

## Discussion

Suspicion and clinical criteria continue to serve as the foundation for VAP diagnosis, however the criteria used to diagnose VAP vary widely, impacting reports of incidence and outcomes. Historically, VAP diagnosis has been based on 2 or 3 components: (1) systemic signs of infection, (2) new or worsening infiltrates seen on chest imaging, and (3) microbiologic evidence of pulmonary parenchymal infection when available [40]. However, the false positive rate is high for clinical symptoms (e.g. fever [42%]), purulent airway secretions (67%), and chest roentenograms [41, 42]. Moreover, combining these criteria does little to improve diagnostic performance [43], and the use of histopathology and microbiology alone carries considerable limitations [21–24, 40].

Numerous diagnostic algorithms have been proposed to standardize the diagnosis, allow for easier identification, and improve inter-study comparability. Patient characteristics in our cohort were largely similar to those of other published cohorts, including age [9, 17, 44–48], male gender predominance [9, 45, 49–53], APACHE II score [45–48, 51, 52, 54], MV duration [49, 52, 54–56], re-intubation rates [9, 52, 57], ICU LOS [47–50, 52, 53, 55], and hospital LOS [47, 50, 52, 55]. In particular, the ICU LOS and mortality were similar to other published VAP cohorts in Iran [53, 58, 59]. Moreover, the array of cultured and MDR pathogens, was consistent with prior studies [51].

A direct comparison of the correlation and diagnostic performance of the VAP algorithms is important for both individual patient care and epidemiology, cross-study comparisons, and meta-analyses. If algorithms have suboptimal sensitivity, specificity, or do not correlate well, subsequent meta-analyses and epidemiologic investigations will be flawed from inception. Direct comparisons of the performance characteristics of the CDC/NHSN, CPIS, HELICS, and the historical Johanson criteria have not previously been reported. Moreover, only two studies were identified that compared VAP diagnostic algorithms [31, 60]. HELICS was chosen as the reference standard due to its wide international and regional use (Europe, Australia, Near- and Middle East [including Iran]), and as it has been used as the reference standard for numerous other studies [2, 35–38, 61]. CDC/NHSN and CPIS criteria were chosen as the other two most widely recognized and used criteria (especially in North America). The Johanson criteria was selected as the third comparator for its historical significance. The sensitivity of the CPIS and Johanson methods was moderate, whereas CDC/NHSN was poor. Moreover, the diagnostic agreement was substantial for CPIS, moderate for Johanson, and only slight for CDC/NHSN (Table 5). Algorithm accuracy was improved by adding serum procalcitonin > 0.5 ng/ml, however, similar to prior reports, the addition of microbiological data to the clinical definitions did not significantly improve the sensitivity or specificity [40].

These findings suggest that combining cohorts based on HELICS and CPIS may be reasonable for meta-analysis or population studies, but the same may not be true for studies based on CDC/NHSN criteria as the diagnostic agreement is poor. Moreover, it is recommended that studies report serum procalcitonin values to better refine their data sets to optimize data utility as diagnostic algorithms evolve to best facilitate future meta-analyses and as procalcitonin may correlate with mortality [62]. Lastly, this data highlights how little progress these complicated VAP diagnostic algorithms have made beyond that of the historical and simple Johanson criteria. These algorithms will most certainly undergo modification, and it is important that investigators clearly define their patient populations and present the data in a way that allows the data to inform future decisions as the diagnostic techniques evolve.

## Limitations

The non-randomized methodology and absence of histopathology confirmation of VAP diagnosis are limitations of this study. This study was performed in a resource-limited setting in a low-to-middle income country (LMIC) and limiting the study cohort to those with ante- or post-mortem histology would have introduced selection bias and served as a barrier for subject recruitment.

The use of TAC specimens is a minor limitation as positive quantitative TAC’s have been reported to have a high degree of correlation with broncho-alveolar lavage in VAP patients and are a useful minimally invasive diagnostic tool [63–65].

Lastly, the serum procalcitonin values were not significantly elevated in the VAP vs. no-VAP group. Procalcitonin is not specific to infection location (i.e. VAP). It may rise with bacterial infections in other locations as well. The no-VAP group did not equate to “no infection anywhere.” Indeed, infections are common in ICU patients ranging from catheter-associated urinary tract infections and other device infections, to soft-tissue infections or even peritonitis from a perforated viscus. There were some patients in the no-VAP group that had non-pulmonary infections with elevated procalcitonin values that raised the mean. It would not be appropriate to remove these patients from the analysis for the following reasons: (1) it would skew remove the real-world applicability of the data, and (2) the study would fall below the necessary sample size required. Lastly, it’s worth noting that procalcitonin values were not a study endpoint and the study was not powered for this purpose.

## Conclusion

Ventilator-associated pneumonia remains a considerable source of morbidity and mortality in modern ICUs. The optimal diagnostic method remains unclear. Using HELICS criteria as the reference standard, CPIS displayed substantial diagnostic agreement whereas CDC/NHSN and Johanson criteria displayed slight and moderate agreement respectively. Accuracy was improved with the addition of serum procalcitonin > 0.5 ng/ml, but not positive quantitative endotracheal aspirate culture. These findings suggest that combining cohorts based on HELICS and CPIS may be reasonable for meta-analysis or population studies, but the same may not be true for studies based on CDC/NHSN criteria.

## Data Availability

All data generated or analyzed during this study are included in this article. De-identified individual subject data may be available from the corresponding author on reasonable.

## Abbreviations

VAP: Ventilator-associated pneumonia
CPIS: Clinical Pulmonary Infection Score
CDC/NHSN: Centers for Disease Control and Prevention’s National Healthcare Safety HELICS Hospital in Europe Link for Infection Control through Surveillance
TP: True positive
FN: False negative
NI: Nosocomial infection
ICU: Intensive care unit
MV: Mechanical ventilation
LOS: Length-of-stay
APACHE: Acute Physiology and Chronic Health Evaluation
SD: Standard deviation
χ^2^: Chi-square test
*J*: Youden’s J Statistic, or Youden Index
MDR: Multiple drug resistant
